# Supplementary material for: Unexpected diversity of CRISPR unveils some evolutionary patterns of repeated sequences in Mycobacterium tuberculosis
Source: BMC Genomics. 2020 Nov 30;21:841. doi: 10.1186/s12864-020-07178-6 (PMC7708916; doi:10.1186/s12864-020-07178-6)

**Supplementary file 3 – Exploration of read numbers for the reconstruction and identification of duplications, the case of ERR718197 (L1.1.1.7).** This table shows the number of reads containing at least the 12 last nucleotides of one spacer at its beginning, followed by a Direct Repeat, followed by at least 12 first nucleotides of one spacer.


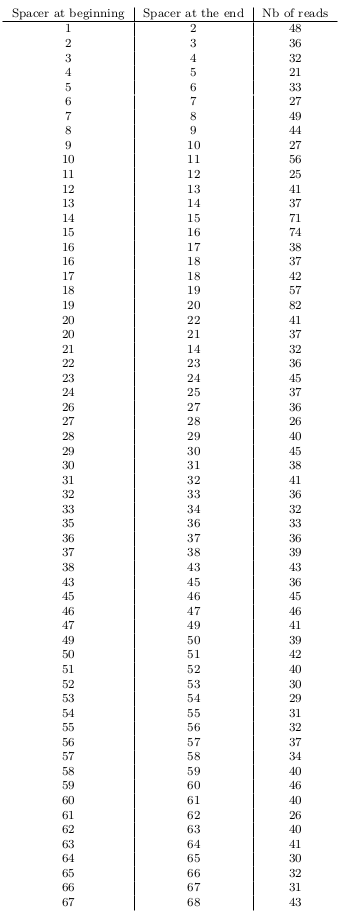

Supplement: Supplementary file 3 — Additional file 3: Supplemental file 3. Exploration of read numbers for the reconstruction and identification of duplications, the case of ERR718197. [file 12864_2020_7178_MOESM3_ESM.docx]
