# Supplementary material for: Unexpected diversity of CRISPR unveils some evolutionary patterns of repeated sequences in Mycobacterium tuberculosis
Source: BMC Genomics. 2020 Nov 30;21:841. doi: 10.1186/s12864-020-07178-6 (PMC7708916; doi:10.1186/s12864-020-07178-6)

**Supplementary File 4 – Confirmation of sp35 presence after spacer 41 in two Sequence runs from strains belonging to L5 and L2 respectively.** A. Reads from SRR998631 (*M. tuberculosis* variant *africanum*) having at least 12 nucl of spacer 41, one DR0 followed by 12 nucl of sp. 35. B. Reads from ERR234248 (L2.1) having at least 12 nucl of spacer 41 and 12 nucl of sp. 35.

A


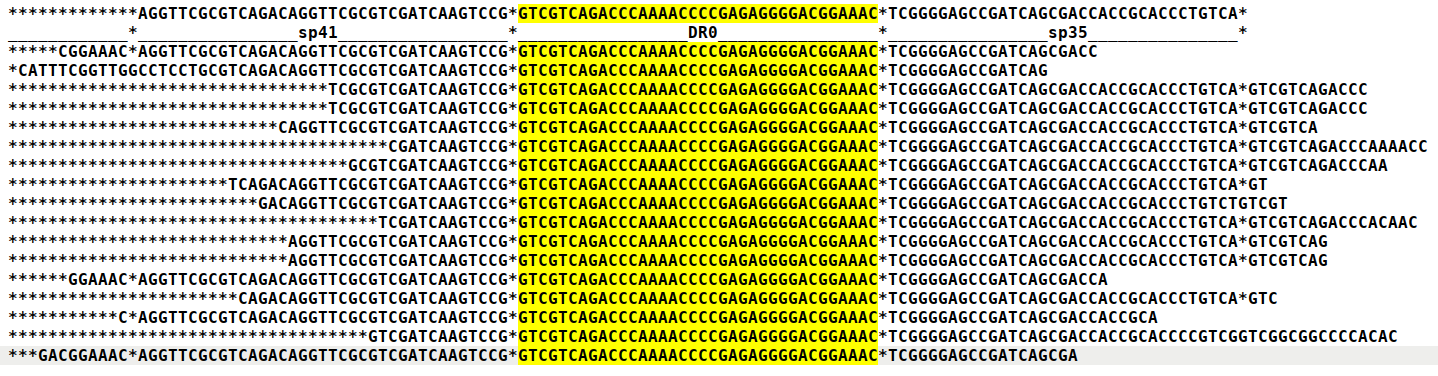


B


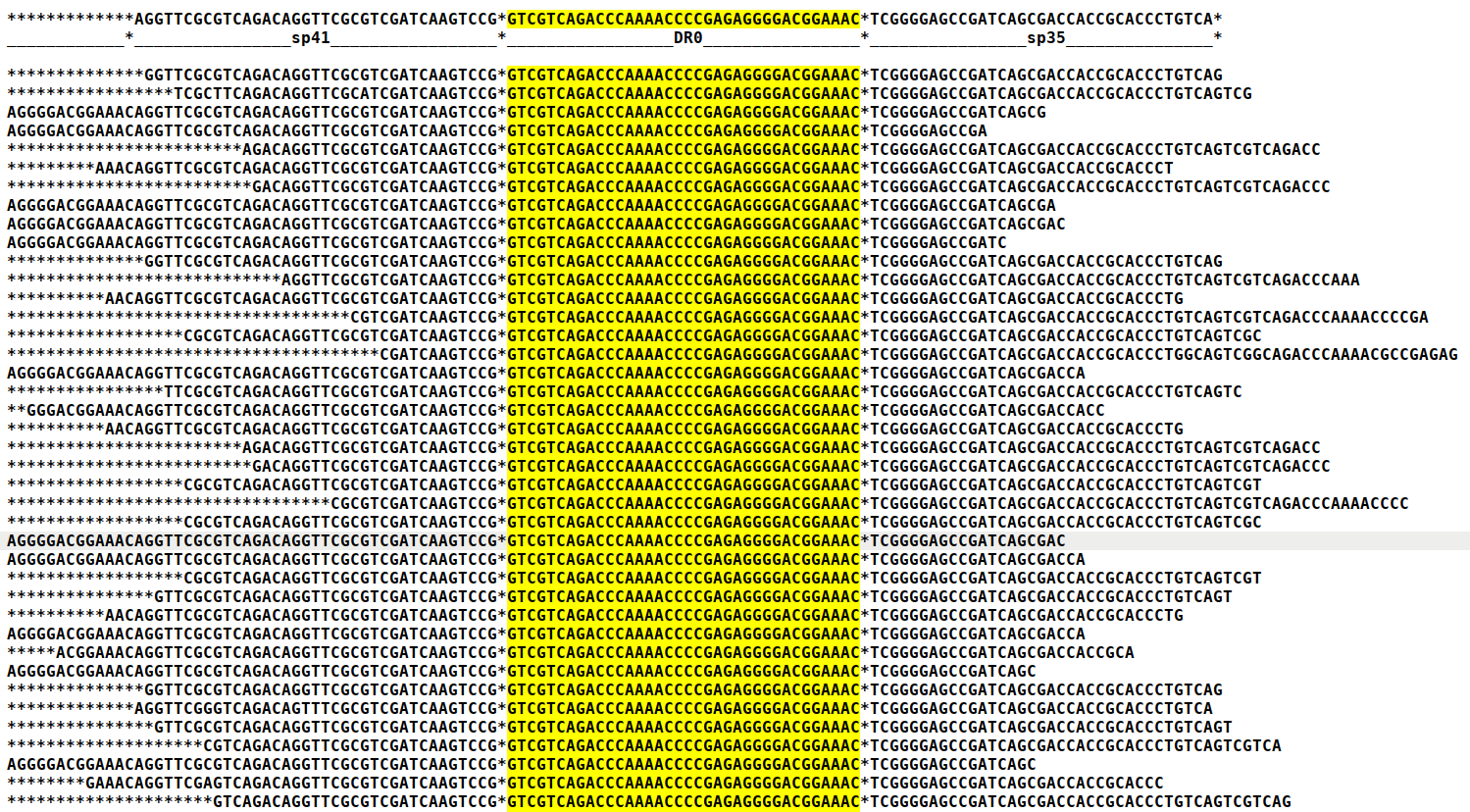

Supplement: Supplementary file 4 — Additional file 4: Supplemental file 4. Confirmation of sp35 presence after spacer 41 in two Sequence runs from clinical isolatess belonging to L5 and L2 respectively [file 12864_2020_7178_MOESM4_ESM.docx]
