# Supplementary material for: Unexpected diversity of CRISPR unveils some evolutionary patterns of repeated sequences in Mycobacterium tuberculosis
Source: BMC Genomics. 2020 Nov 30;21:841. doi: 10.1186/s12864-020-07178-6 (PMC7708916; doi:10.1186/s12864-020-07178-6)
Supplement: Supplementary file 5 — Additional file 5: Supplemental file 5. Spacer 4, spacer 6 and spacer 38 variants in parallel with 43-spacers spoligotyping probes [file 12864_2020_7178_MOESM5_ESM.docx]

**Supplementary file 5 - Spacer 4, spacer 6 and spacer 38 variants in parallel with 43-spacers spoligotyping probes.**

1. **Spacer 4**

>most frequent sequence (esp4)

TCGCAAGCGCCGTGCTTCCAGTGATCGCCTTCTA

>variant 1 (esp4(2)) [found in all L6 strains]

TCGCAAGCGCCGTGCTTCCAGTGATC**A**CCTTCTA

>variant 2 (esp4(1)) [found in some L6 strains]

TCGCAAGCGCCGTGCTTCCAGTGATC**A**CCTT**G**TA

Spoligo43 probe #3

3…………... CCGTGCTTCCAGTGATCGCCTTCTA

1. **Spacer 6**

> most frequent sequence (esp6)

ATGTGCGCCGTCGCCGTAAGTGCCCCACGGCCCGT

> variant 1 (esp6(1))

ATGTGCGCCGTCGCCGTAAGT**A**CCCCACGGCCCGT

> variant 2 (esp6(2))

ATGTGCGCCGTCGCCGTAAGT**A**CCCCACGGCC**A**GT

1. **Spacer 38**

>esp38

TGCCCCGGCGTTTAGCGATCACAACACCAACTAATG

>esp38(1) [found in L1.1.1 strains]

TGCCCC**A**GCGTTTAGCGATCACAACACCAACTAATG

Spoligo43 probe #28

28 .TGCCCCGGCGTTTAGCGATCACAAC
