# Supplementary figures and images for: Unexpected diversity of CRISPR unveils some evolutionary patterns of repeated sequences in Mycobacterium tuberculosis
Source: BMC Genomics. 2020 Nov 30;21:841. doi: 10.1186/s12864-020-07178-6 (PMC7708916; doi:10.1186/s12864-020-07178-6)

## Slide 1
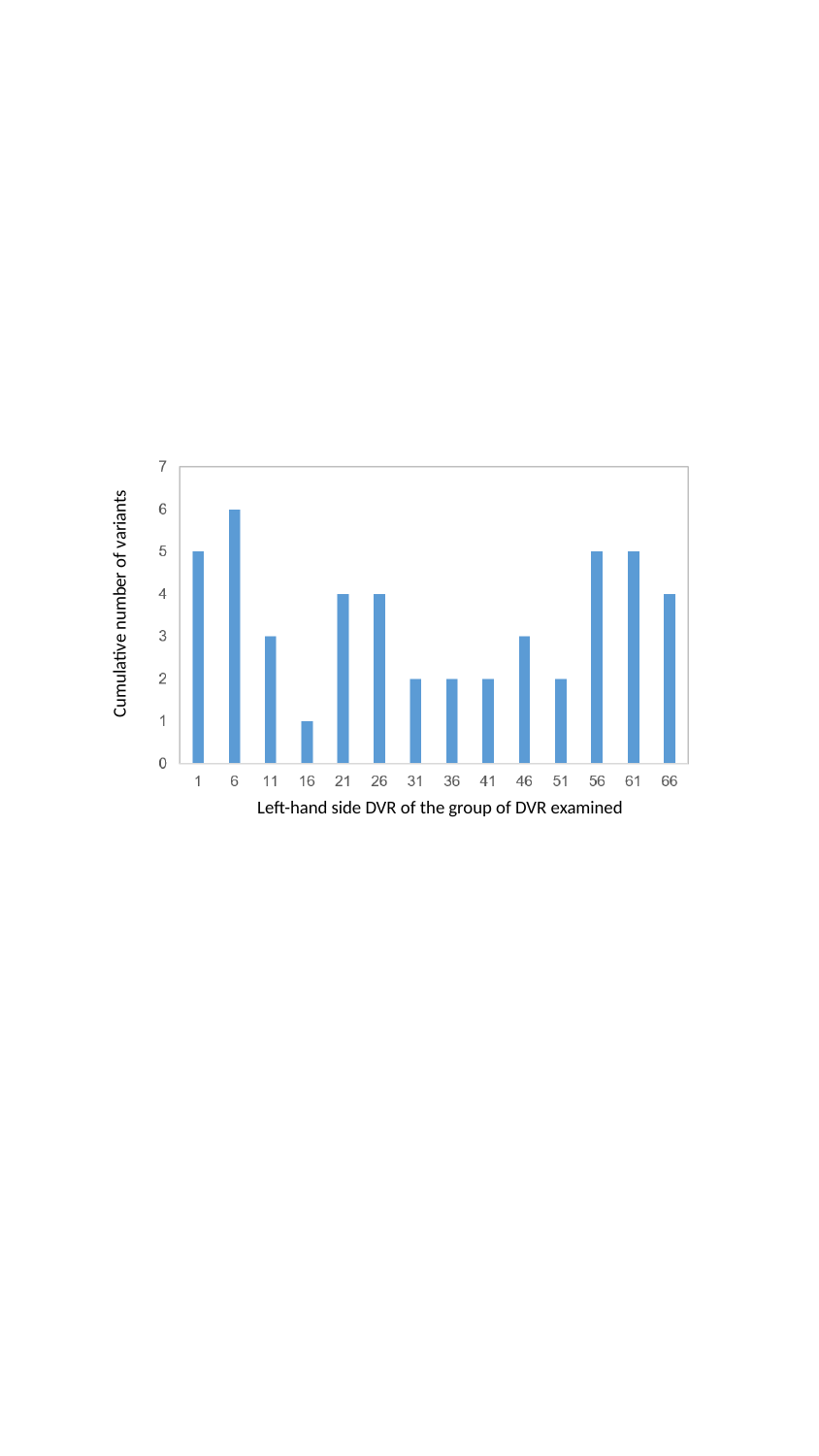

Cumulative number of variants
Left-hand side DVR of the group of DVR examined

Supplement: Supplementary file 6 — Additional file 6: Supplemental file 6. Cumulative punctual variant numbers 5DR variants + spacer variants) in groups of 5 successive DVR from DVR1–5 to the last three DVR (DVR66–68) [file 12864_2020_7178_MOESM6_ESM.pptx]
